# A Paradigm Shift in the Combination Changes of SARS-CoV-2 Variants and Increased Spread of Delta Variant (B.1.617.2) across the World

**DOI:** 10.14336/AD.2021.1117

**Published:** 2022-06-01

**Authors:** Chiranjib Chakraborty, Ashish Ranjan Sharma, Manojit Bhattacharya, Govindasamy Agoramoorthy, Sang-Soo Lee

**Affiliations:** ^1^Department of Biotechnology, School of Life Science and Biotechnology, Adamas University, Kolkata, West Bengal 700126, India.; ^2^Institute for Skeletal Aging & Orthopaedic Surgery, Hallym University-Chuncheon Sacred Heart Hospital, Gangwon-do, Korea.; ^3^Department of Zoology, Fakir Mohan University, Vyasa Vihar, Odisha, India.; ^4^College of Pharmacy and Health Care, Tajen University, Pingtung, Taiwan

**Keywords:** Delta variants, SARS-CoV-2, combination of variants, paradigm shifts

## Abstract

Since September 2020, the SARS-CoV-2 variants have gained their dominance worldwide, especially in Kenya, Italy, France, the UK, Turkey, Indonesia, India, Finland, Ireland, Singapore, Denmark, Germany, and Portugal. In this study, we developed a model on the frequency of delta variants across 28 countries (R^2^= 0.1497), displaying the inheritance of mutations during the generation of the delta variants with 123,526 haplotypes. The country-wise haplotype network showed the distribution of haplotypes in USA (10,174), Denmark (5,637), India (4,089), Germany (2,350), Netherlands (1,899), Sweden (1,791), Italy (1,720), France (1,293), Ireland (1,257), Belgium (1,207), Singapore (1,193), Portugal (1,184) and Spain (1,133). Our analysis shows the highest haplotype in Europe with 84% and the lowest in Australia with 0.00001%. A model of scatter plot was generated with a regression line which provided the estimated rate of mutation, including 24.048 substitutions yearly. Our study concluded that the high global prevalence of the delta variants is due to a high frequency of infectivity, supporting the paradigm shift of the viral variants.

This study thoroughly analyzed different variants of SARS-CoV-2 that emerged during the last few months [1-3]. Mutations in the structural and non-structural proteins of SARS-CoV-2 have created diverse variants rapidly. Mutations in S-glycoprotein lead to different properties in the variants that include immune escape, vaccine escape, and neutralizing antibody (nAb) escape [4, 5]. The mutation-triggered escape properties in the variants appear to hinder the therapeutics from combating the pandemic. Scientists have derived the genomic mutation rate of the virus in humans from sequencing viral data retrieved from public repositories. Higher rates of mutations are observed frequently in RNA viruses such as SARS, MERS, allowing the hosts to adapt to the virus [6, 7]. SARS-CoV-2 is more prone to mutations [8, 9], and positive selection was reported in most of the significant mutations facilitating the rapid evolution of the virus [10-12] with about 0.8-2.38×10^-3^ nucleotide substitutions/site per year [13]. The positive selection of some significant mutations favors the process of natural selection in the case of some viral variants. Recently, 11 variants of SARS-CoV-2 were reported that include the alpha, gamma, beta, epsilon, delta, delta plus, theta, eta, iota, lambda, and kappa. Among them, some are VOCs or VOIs, and supplementary Table 1 shows the lineages of variants. Nonetheless, it is necessary to comprehend the significance of mutations in the SARS-CoV-2 variants and their molecular machinery [14]. When the delta variant was detected in India a few months ago, it created a catastrophic second wave that killed over 300,000 people [15, 16].

The emergence of the new delta variant (B.1.617.2) has made the pandemic more critical across countries [17]. The first report on the isolation of the delta variant was documented in Maharashtra, India [18]. Subsequently, it was isolated across 60 countries. In the USA, the Oklahoma and Colorado states have reported the outbreak of the variant recently [19, 20]. It also spread across the EU, including Finland [21]. Australia fought against the delta variant to neutralize the catastrophic effect during the pandemic [22]. At the same time, there is a report of isolation of the variant of SARS-CoV-2 from the Guangzhou region, China [23]. The delta variant currently dominates the world, leading to the following queries: Does natural selection favor the delta variant? Is the variant part of positive selection?

Researchers are analyzing the mutation of variants to demonstrate positive selection [11]. Some significant mutations were observed in India during the second wave inside RBD regions of S-glycoprotein (T478K, L452R, and E484Q) and outside of the RBD region (D614G and P681R) [18, 24]. In addition, other mutations such as G142D, T19R, Δ156-157, R158G, and D950N are also reported [24]. Baral et al. have illustrated the mutations in RBD regions, and these mutations (T478K/ L452R) can alter the structure in the receptor-binding interface converting the binding affinity. Finally, these structural changes in RBD can modify the interfaces of receptor and antibody-binding involved in the immune evasion phenomena [25]. Therefore, comprehending the significant mutations is vital to evaluate the spread of the delta variants.

Neutralizing antibodies (nAbs) protect against SARS-CoV-2, and scientists have reported that the delta variant is associated with the escape phenomena of nAb or vaccines [26]. Planas et al. observed the delta variant showing resistance to neutralization through the monoclonal antibodies such as anti-RBD and some anti-NTD related monoclonal antibodies [27]. Different studies observed that E484Q is a more proficient mutation to evade antibody neutralization than T478K in the variant [28-30]. These two mutations are noted in the delta variant. However, the spread of the delta variant might extend the pandemic period and negatively affect the current vaccination program in different countries.

This study analyzed the composition of SARS-CoV-2 variants with country-wise haplotype and scatter plot analysis of all lineages to understand the mutational landscape. Also, country-wise frequency analysis of the delta variant was done to illustrate the demographic-specific composition of the variants in the UK, USA, Brazil, India, and France, where the delta variant was dominant. Furthermore, this study observed the paradigm shifts in the composition of SARS-CoV-2 variants and signature shifts towards the delta variants. Our study concludes that the rapid spread of the delta variant globally is due to its increased frequency of infectivity, showing the paradigm shift of this variant.

**Figure 1.**
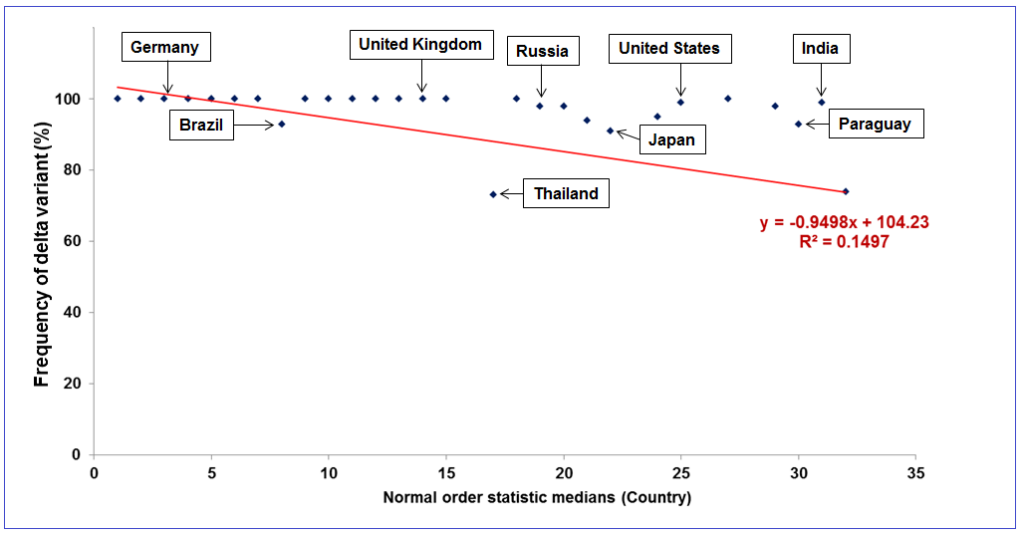
A statistical model illustrates the frequency of delta variants from 28 countries.

## MATERIALS AND METHODS

### Data collection

The latest information on the literature was accessed through the Web of Science [31], PubMed [32, 33], and Google Scholar [34]. The study collected/retrieved data on the delta variants from the CDC [35] and WHO [36]. Keywords such as “delta variants” and “B.1.617.2” were used to locate data, and the genomic data were retrieved from the GISAID database [37, 38]. The COVID-19 data were gathered from several countries and also from Nextstrain [39-42].

### Data analysis and data interpretation

The study used servers, such as Nextstrain (SARS-CoV-2 resources) [41, 42], CoVariants [38,43] and 2019 Novel Coronavirus Resource (2019nCoVR) (developed by CNCB) [44]. The haplotype network of delta variants was constructed using the MUSCLE algorithm [45], VEP algorithm [46], and 2019nCoVR [44]. Delta variant spread frequency model was generated by a statistics software (PAST 4.03 software) [47]. MATLAB was used to analyze and plot graphs [48].

## RESULTS

### The composition of SARS-CoV-2 variants and the share of analyzed sequences in the last two weeks

All collected samples of SARS-CoV-2 for genome sequencing are sequenced from different countries and submitted to GISAID. We have analyzed the share of SARS-CoV-2 different variants’ sequences in the last two weeks. We found that most of the sample sequences were identified as delta variants, and the collected sample sequence has shown 100% delta variants in South Africa, Mexico, Russia, Pakistan, Canada, Singapore, Spain, Portugal, UK, Germany, USA, Italy and Turkey. At the same time, sample sequences from France have shown 99% frequency. The sample sequence from Japan and India has shown 98% delta variants, while Brazil showed 96%. Similarly, the sample sequence from Thiland was 92% (Supplementary Fig. 1A). The study showed that the delta variant share started to increase after February 24, 2021. However, Turkey's fluctuation in delta variant share finally achieved 100% in September 2021 (Supplementary Fig. 1B).

**Figure 2.**
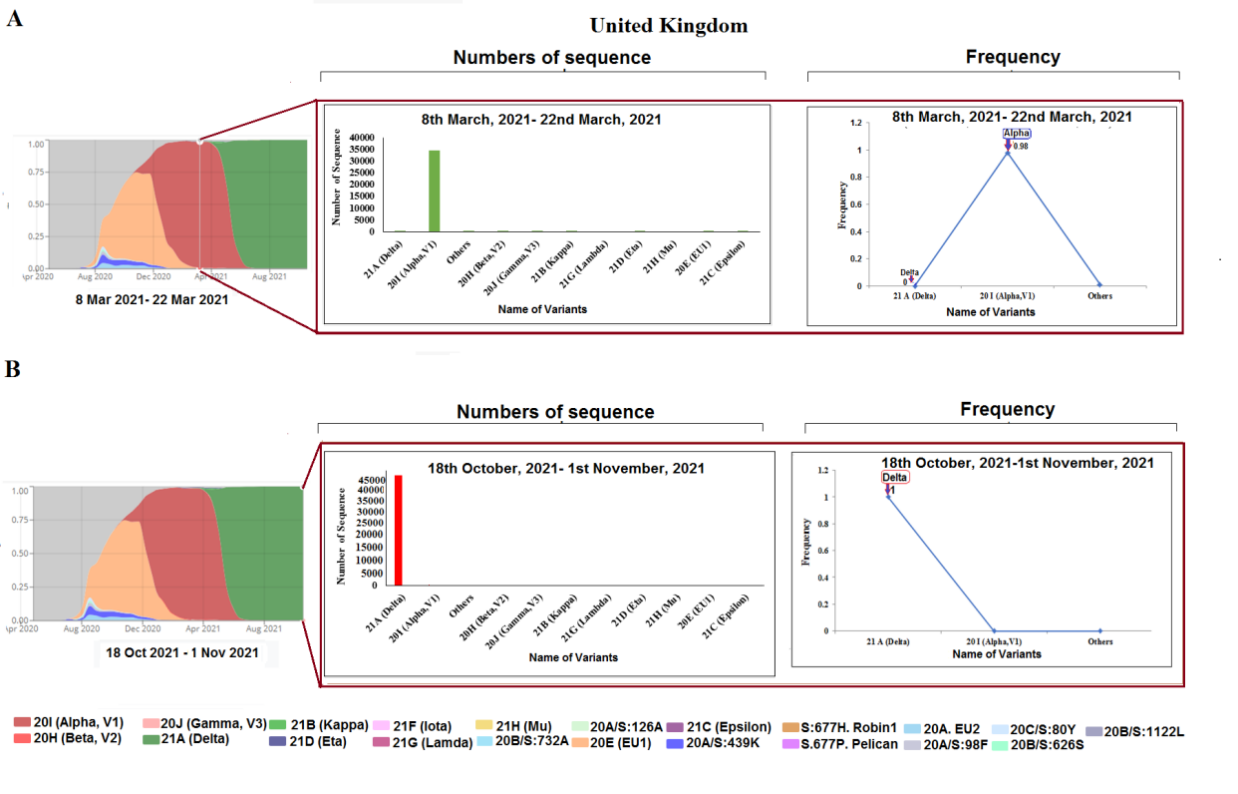
Composition changes of the SARS-CoV-2 variants in the UK. (A) Comparison of SARS-CoV-2 variants composition recorded from March 8 to March 22, 2021, in the UK. (B) Comparison of SARS-CoV-2 variants composition recorded from Ocotober 18 to November 1, 2021, in the UK.

**Figure 3.**
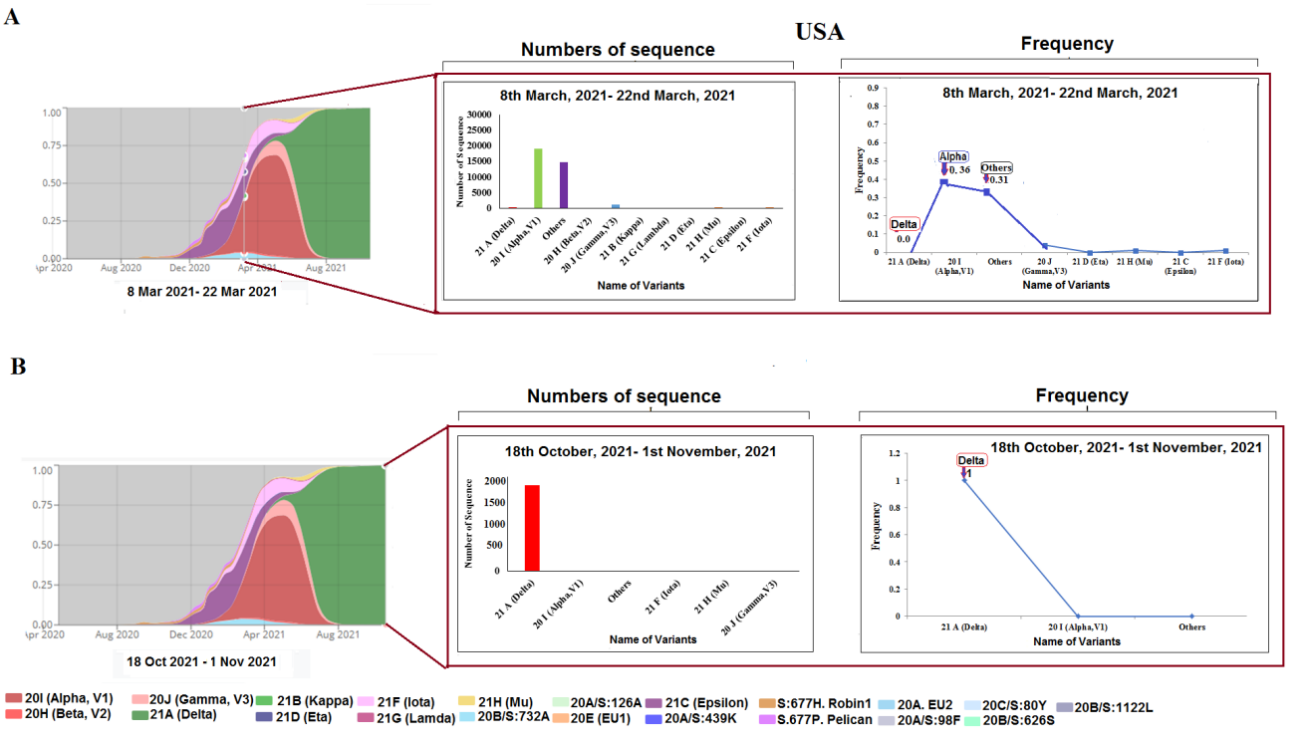
SARS-CoV-2 variants composition change in the USA. (A) Comparison of SARS-CoV-2 variants composition recorded from March 8 to March 22, 2021, in the USA. (B) Comparison of SARS-CoV-2 variants composition recorded from Ocotober 18 to November 1, 2021, in the USA.

**Figure 4.**
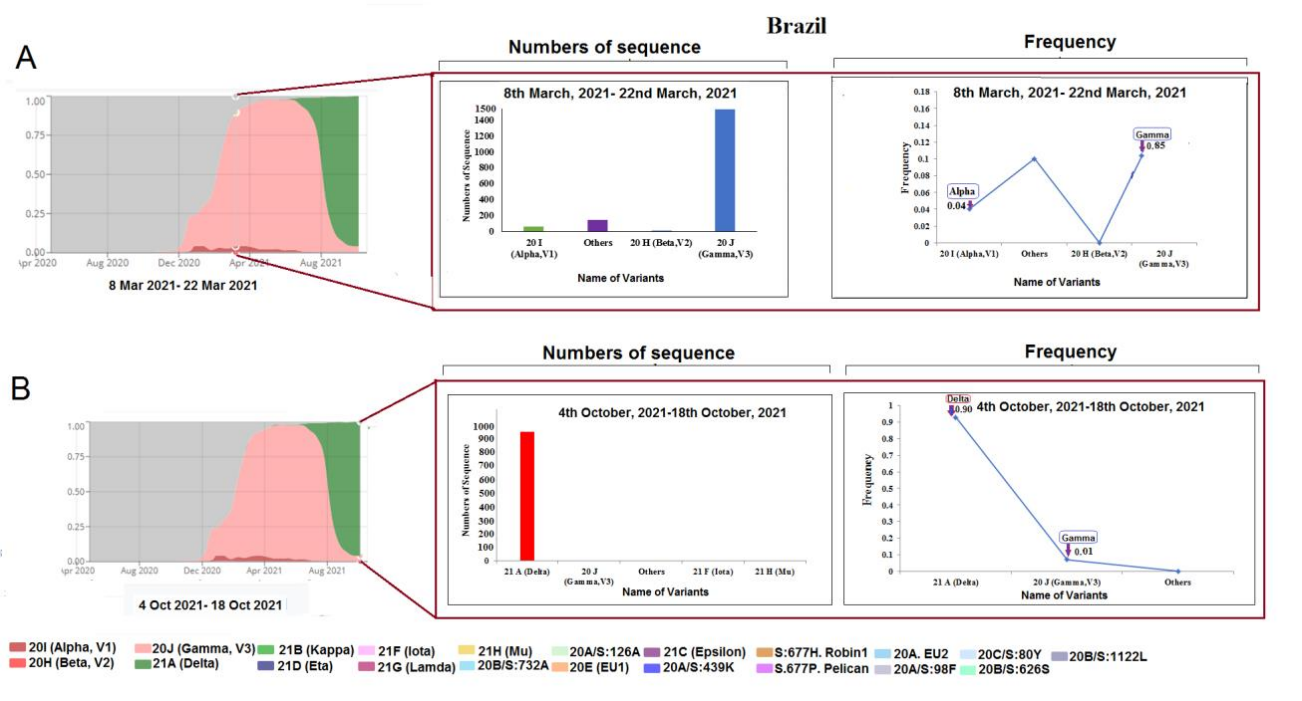
SARS-CoV-2 variants composition change in Brazil. (A) Comparison of SARS-CoV-2 variants composition recorded from March 8 to March 22, 2021, in Brazil. (B) Comparison of SARS-CoV-2 variants composition recorded from October 4 to October 18, 2021, in Brazil.

**Figure 5.**
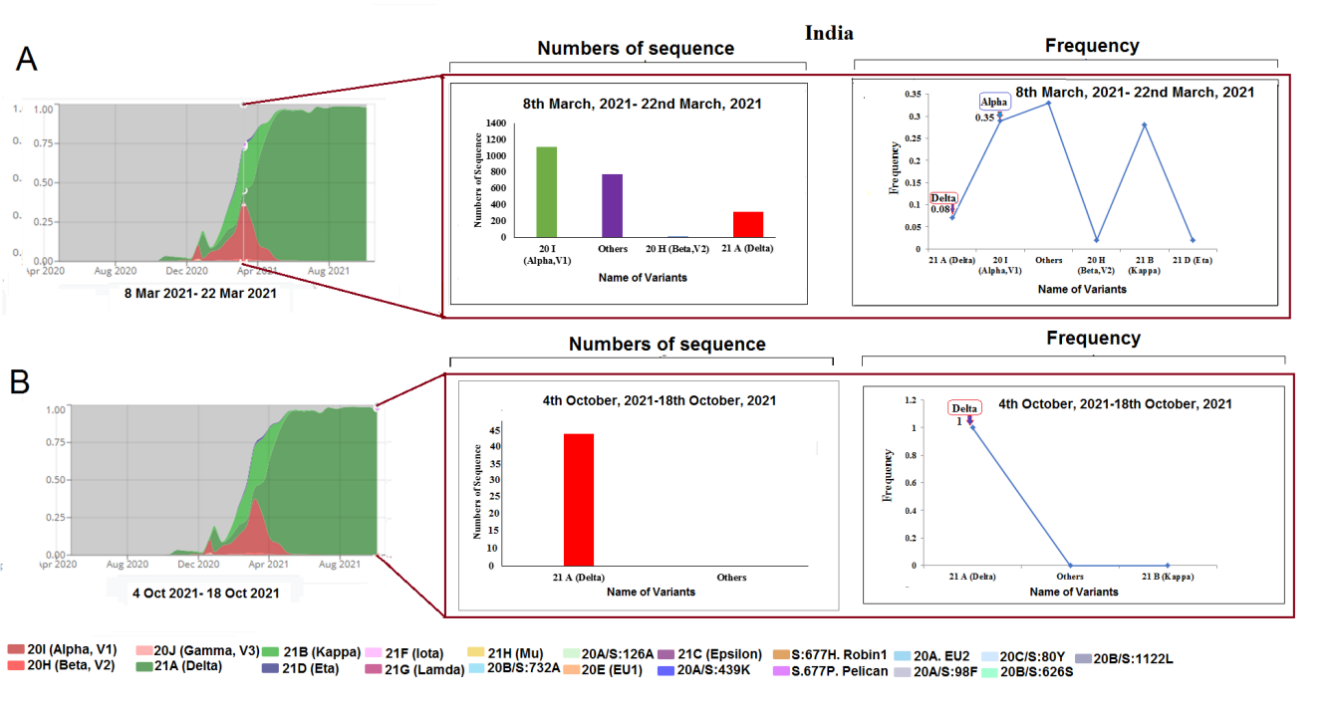
SARS-CoV-2 variants composition change in India. (A) Comparison of SARS-CoV-2 variants composition recorded from March 8 to March 22, 2021, in India. (B) Comparison of SARS-CoV-2 variants composition recorded from October 4 to October 18, 2021, in India.

**Figure 6.**
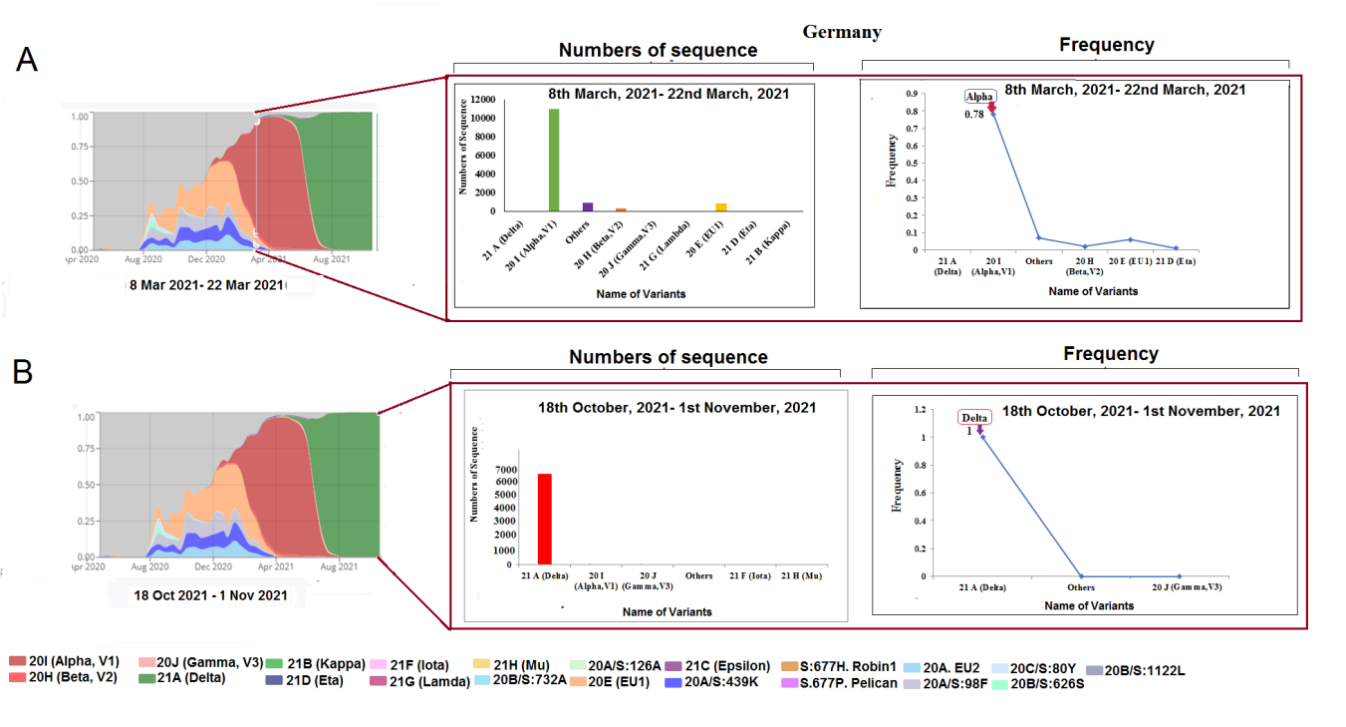
SARS-CoV-2 variants composition change in Germany. (A) Comparison of SARS-CoV-2 variants composition recorded from March 8 to March 22, 2021, in Germany. (B) Comparison of SARS-CoV-2 variants composition recorded from Ocotober 18 to November 1, 2021, in Germany.

### Country-wise spread frequency analysis of delta variant

The country-wise spread frequency of the variants has shown 80% frequency or more, and it achieved more than 80% status during June-July 2021 in the UK (Supplementary Fig. 2). Similarly, delta variant spread frequency was about 100% (approximately) in June-July 2021 (USA, Germany and Ireland), August 2021 (Singapore), July-August 2021 (Slovakia), September-October 2021 (Supplementary Fig. 2). Again, the variant spread frequency was observed over 80% in Sweden, Russia, Switzerland, South Korea, Canada, Israel, South Africa, India, France and Brazil (Supplementary Fig. 2). We have developed a model of the frequency of delta variants spread in different countries (Fig. 1). Interestingly, it was observed from the spread frequency estimation of the delta variant of different countries that the variant was noted very early in India during October 26, 2020 (Supplementary Fig. 1B and Fig. 2G). However, the frequency was significantly less, about 0.65%. It was the first spotted spread frequency of delta variant among all courtiers throughout the world. The instance confirms the origin of the delta variant in India.

### The demographic-specific composition of SARS-CoV-2 variants and observed delta variant dominance

We have analyzed the composition of the SARS-CoV-2 variants country-wise on different days of 2021. Our analysis shows the composition pattern of the SARS-CoV-2 variants.

**Figure 7.**
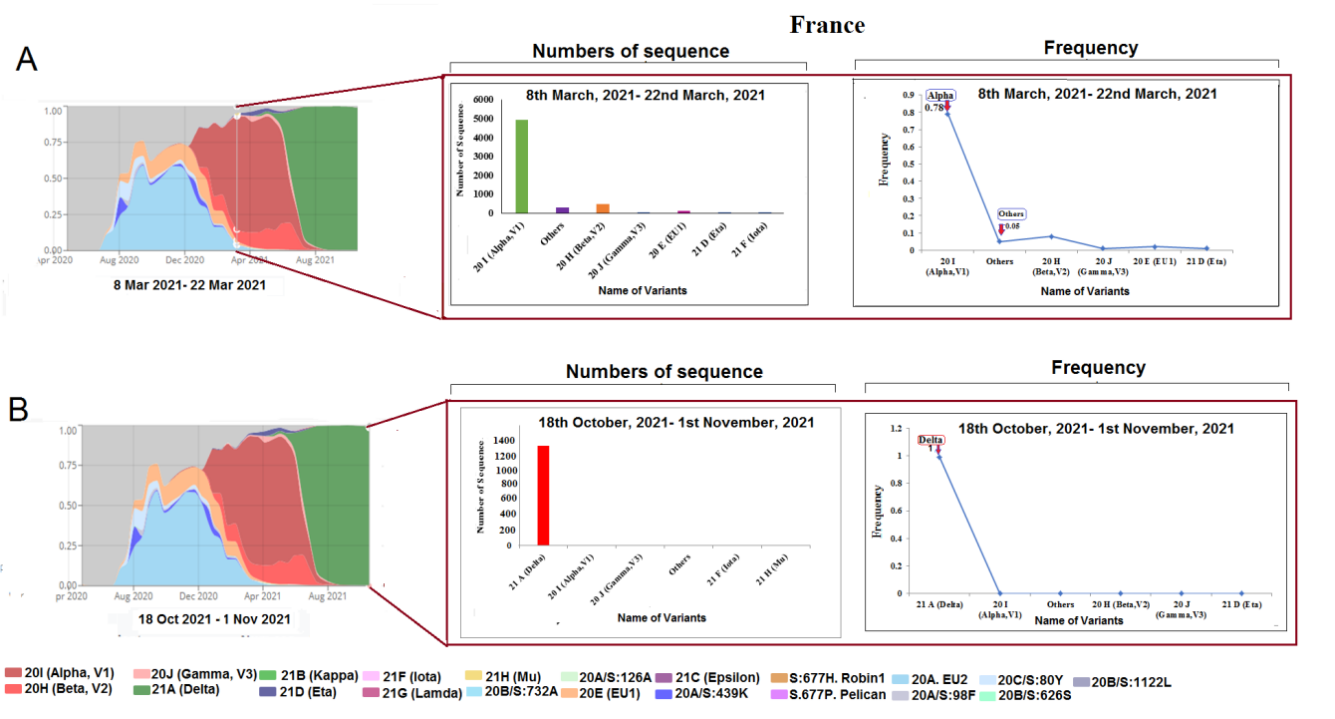
SARS-CoV-2 variants composition change in France. (A) Comparison of SARS-CoV-2 variants composition recorded from March 8 to March 22, 2021, in France. (B) Comparison of SARS-CoV-2 variants composition recorded from from Ocotober 18 to November 1, 2021, in France.

### Change in the composition of SARS-CoV-2 variants in the UK

The first analysis of the composition of SARS-CoV-2 variants was studied in the UK. Our study observed the combination of SARS-CoV-2 variants in the UK at different time points in a week's time. For example, from March 8 to March 22, 2021, and October 18 to November 1, 2021. We analyzed the number of alpha (B.1.1.7 lineage) and delta variant sequences from the sampling. Results showed more or less 34495 sequences for the alpha variant and five delta variants from March 8 to March 22, 2021 (Fig. 2A). At the same time, we noted one sequence for the alpha, five other variant and more or less 49,725 delta sequences from October 18 to November 1, 2021 (Fig. 2B). The changed frequency of both the alpha and delta variants was also observed. In this direction, our study observed the change frequency of the alpha variant as 0.98 (March 8 to March 22, 2021) and 0.00 (October 18 to November 1, 2021). Similarly, the change frequency of delta variants was 0.00 (March 8 to March 22, 2021) and 1.00 (October 18 to November 1, 2021). Our study observed a composition shift in the SARS-CoV-2 variants. It marks a prototype shift from the alpha variant to delta variant in the UK, and the pattern demonstrates a signature of positive selection of the delta variant.

**Figure 8.**
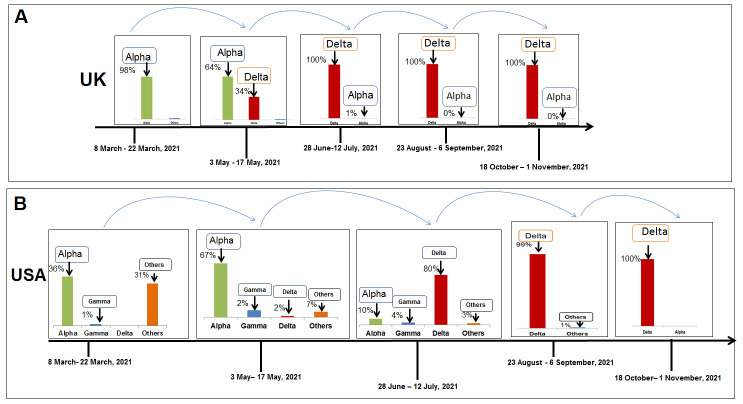
A model that illustrates the paradigm shifts in the composition of SARS-CoV-2 lineages and increases the delta variant in the variant’s landscape noted in UK and USA. (A) Signature change in the composition of SARS-CoV-2 lineages and increases the delta variant in the UK. (B) Paradigm shifts in the composition of SARS-CoV-2 lineages and increases the delta Variant in the USA.

### Alteration in the composition of SARS-CoV-2 variants in the USA

We analyzed the composition of SARS-CoV-2 variants in the USA at different times: two weeks (from March 8 to March 22, 2021, and from October 18 to November 1, 2021). Several variant sequences from the sample analysis using open-source data were identified. Moreover, approximately 18,724 sequences for alpha variants were observed as there were 16,339 others variants, seven delta variants from March 8 to March 22, 2021 (Fig.3A). The analysis showed five sequences for other variants, one sequence of gamma variant and more or less 17,052 sequences for delta variant from October 18 to November 1, 2021 (Fig. 3B), and the changes in the frequency of alpha variants were 0.36 (8-22 March 2021) and 0.00 (October 18 to November 1, 2021). Similarly, the changes in the frequency of delta variants were 0.00 (8-22 March 2021) and 1.00 (October 18 to November 1, 2021). This shows that the signature change in the selection of variants appears to be influenced by natural selection.

### Change in the composition of SARS-CoV-2 lineages in Brazil

During the composition analysis of SARS-CoV-2 lineages in Brazil, several significant variants of SARS-CoV-2 (gamma P.1 lineage and alpha B.1.1.7 lineage) were noted. Drastic changes of SARS-CoV-2 lineages during 8-22 March 2021 (Fig. 4A) and October 4 to October 18, 2021, were noted. Our analysis showed that the delta variant in Brazil replaced the gamma, alpha, and other variants. For example, for the gamma variant (1531 sequences), alpha variants (75 sequences), and other variants (190 sequences) were noted during the collection of sample sequences during 8-22 March 22, 2021. Similarly, we noted 945 delta variants and 17 gamma variants from 4-18 October 2021 (Fig. 4B), reducing the number of deposition sequences of the variants. During this period, delta variants (0.90) and alpha variants (0.01) were prominent.

**Figure 9.**
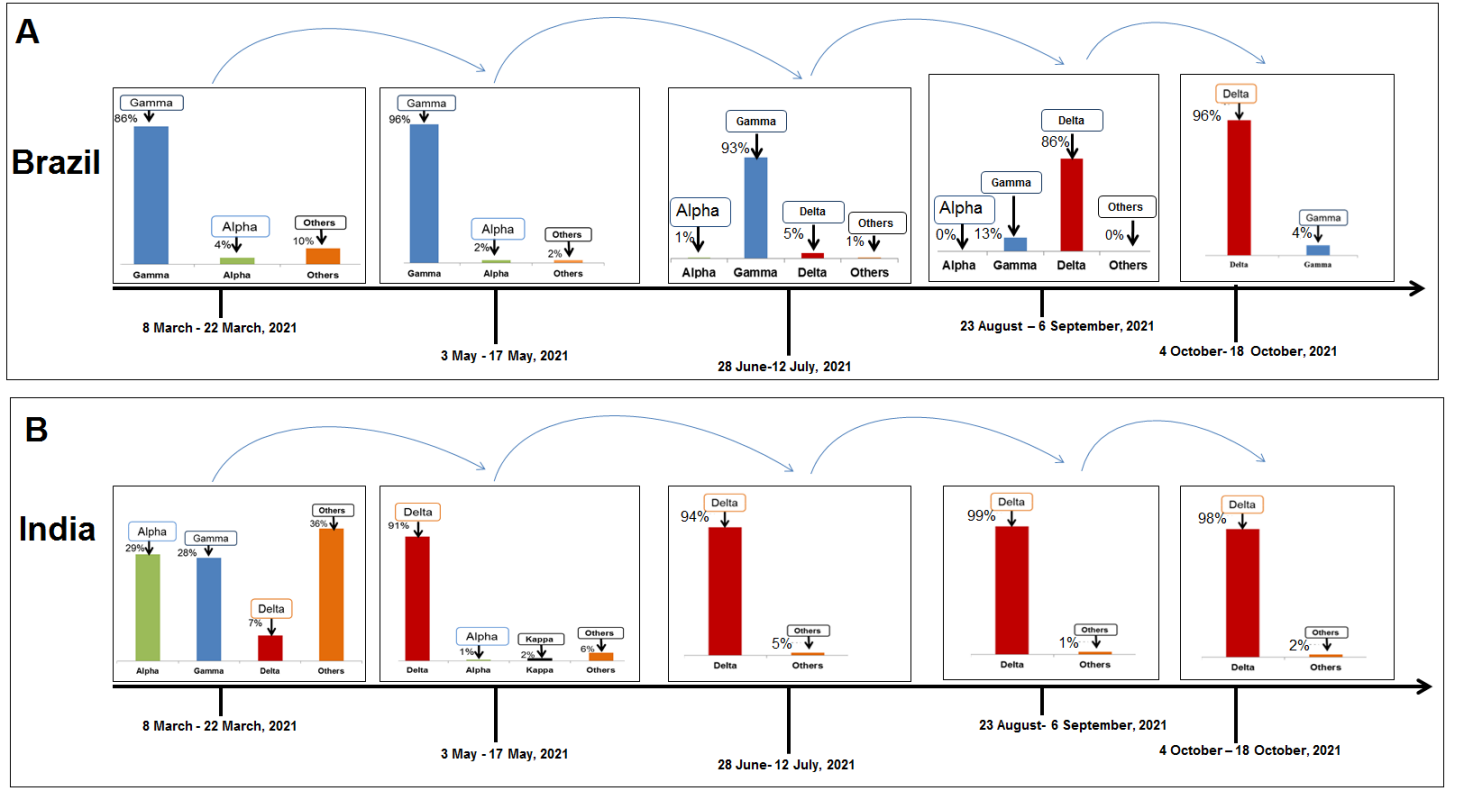
A model that illustrates the paradigm shifts in the composition of SARS-CoV-2 lineages and increases the delta variant in the variant’s landscape noted in Brazil and India. (A) Paradigm shifts in the composition of SARS-CoV-2 lineages and increases the delta variant in Brazil. (B) Paradigm shifts in the composition of SARS-CoV-2 lineages and increases the delta variant in India.

### A shift of the prototype of SARS-CoV-2 lineages in India

We analyzed the composition of SARS-CoV-2 lineages from India and found the alpha, beta, kappa (B.1.617.1 lineage), and eta variants (B.1.525 lineage) been replaced by the delta variant. Together, the frequencies of these variants were: 0.35 for alpha, 0.29 for kappa, 0.08 for delta, and 0.25 for other variants. Our analysis also showed 1196 sequences for alpha, 961 for kappa, 261 for delta, and 850 for other variants during 8-22 March 2021 (Fig. 5A). However, 46 sequences of delta during 4-18 October 2021 were also observed (Fig. 5B). Similar to Brazil, a decrease in the number of samples was noted. Simultaneously, the frequencies for the two variants (1.00 for delta) and none for alpha were detected.

### Change in the composition of SARS-CoV-2 lineages in Germany

A similar pattern was noted with an increase in the delta variant in Germany since May 2021. Initially, 11,395 sequences of alpha variant, 858 of EU1, 353 of beta, and 899 of other variants lasted during 8-22 March 2021 (Fig. 6A). Simultaneously, more or less 6869 sequences of the delta, one for EU1 variant, were noted during October 18 to November 1, 2021 (Fig. 6B). Similar to India, there was a decline in the number of samples with a frequency of 1.00 for delta variant and none for alpha.

### Alteration in the prototype of SARS-CoV-2 lineages in France

In France, we observed a similar situation, and the analysis showed 4985 sequences of alpha, 145 of EU1, 266 of EU2, 495 of beta, and 305 sequences of other variants lasting from 8 to March 22, 2021 (Fig. 7A). Frequency-wise, the variants were distributed as 0.79 of alpha, 0.02 of EU1, 0.04 of EU2, 0.08 of beta, and 0.07 other variants. Simultaneously, the analysis showed 1344 sequences of delta, and five others sequence during October 18 to November 1, 2021 (Fig. 7B). Similar to other countries, our analysis observed a severe decline in sample size with an observed frequency of variants (1.00 for delta and none for other variants).

### The composition of SARS-CoV-2 variants and the variant’s signature shift towards the delta variant

**Figure 10.**
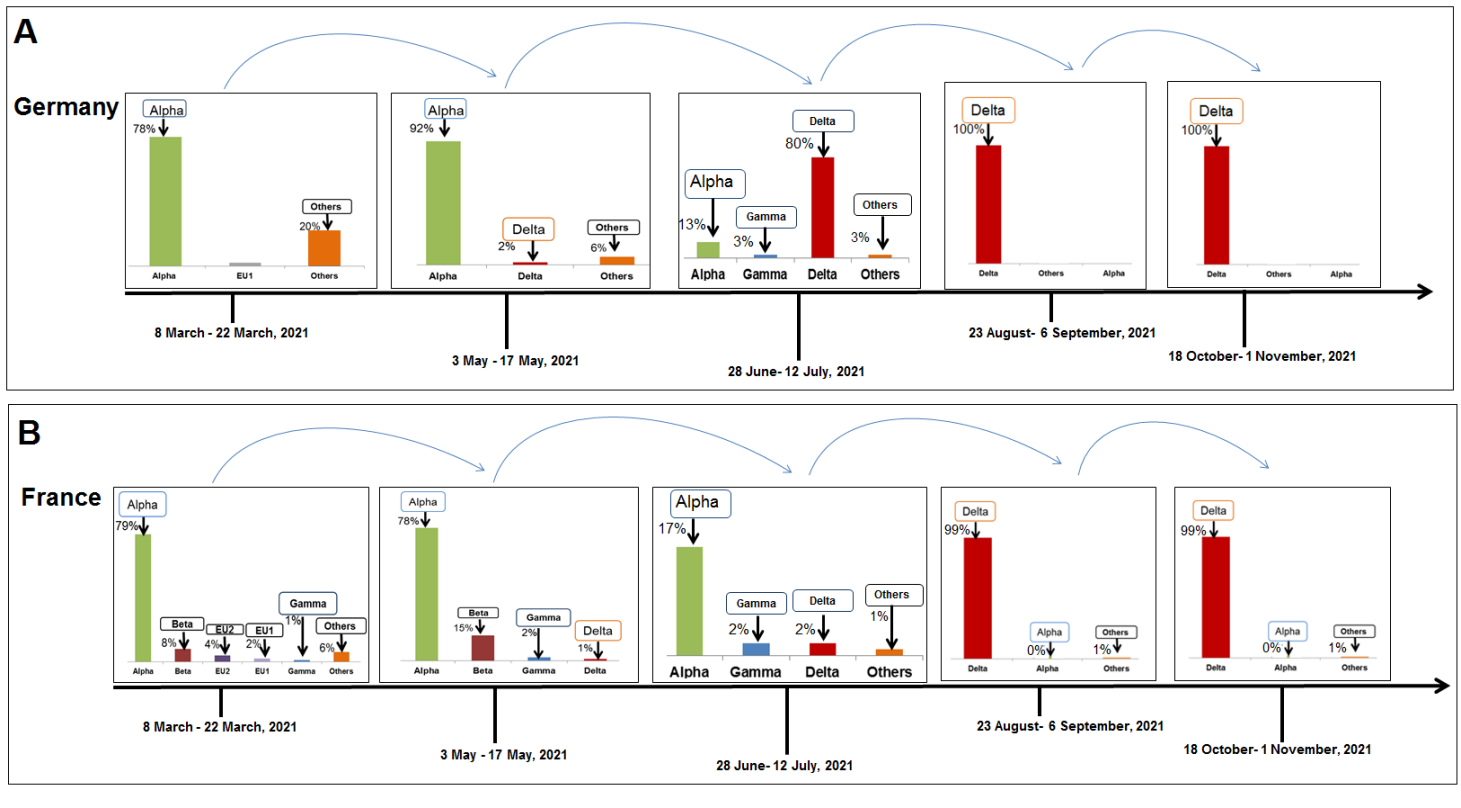
A model that illustrates the paradigm shifts in the composition of SARS-CoV-2 lineages and increases the delta variant in the variant’s landscape noted in Germany and France. (A) Paradigm shifts in the composition of SARS-CoV-2 lineages and increases the delta variant in the Germany. (B) Paradigm shifts in the composition of SARS-CoV-2 lineages and increases the delta variant in the France. The model demonstrates the delta variant takes over the space of the other variants.

To illustrate the entire landscape of paradigm shifts in the composition of variants, we analyzed the composition of SARS-CoV-2 lineages in different countries in the five time points. The landscape of paradigm shifts in the composition of variants in the UK shows the paradigm shift of the alpha variant to the delta variant (Fig. 8A). In the USA, we found the paradigm shift of the alpha, gamma, and other variants to the delta variant (Fig. 8B). Similar patterns were observed in different countries, and the change in different variants to the delta was recorded in Brazil (Fig. 9A), India (Fig. 9B), Germany (Fig. 10A), and France (Fig. 10B). Kirola was the first to find the emergence of this variant transmitted to different countries [49]. Due to the low frequency of the variant, the variant might be indicated as VOI by WHO [2]. However, the increasing frequency was noted afterward. Then, the variant might be marked as VOC [3]. Finally, we observed paradigm shifts in the composition of SARS-CoV-2 variants in different countries. We found an increase of delta variants and a lowering of other variants. Simultaneously, the rise of the delta variant might cause a decrease in different variants. Finally, the dominance of the delta variant was observed in every country.

### Transmission pattern and geographical distribution pattern of delta variant

**Figure 11.**
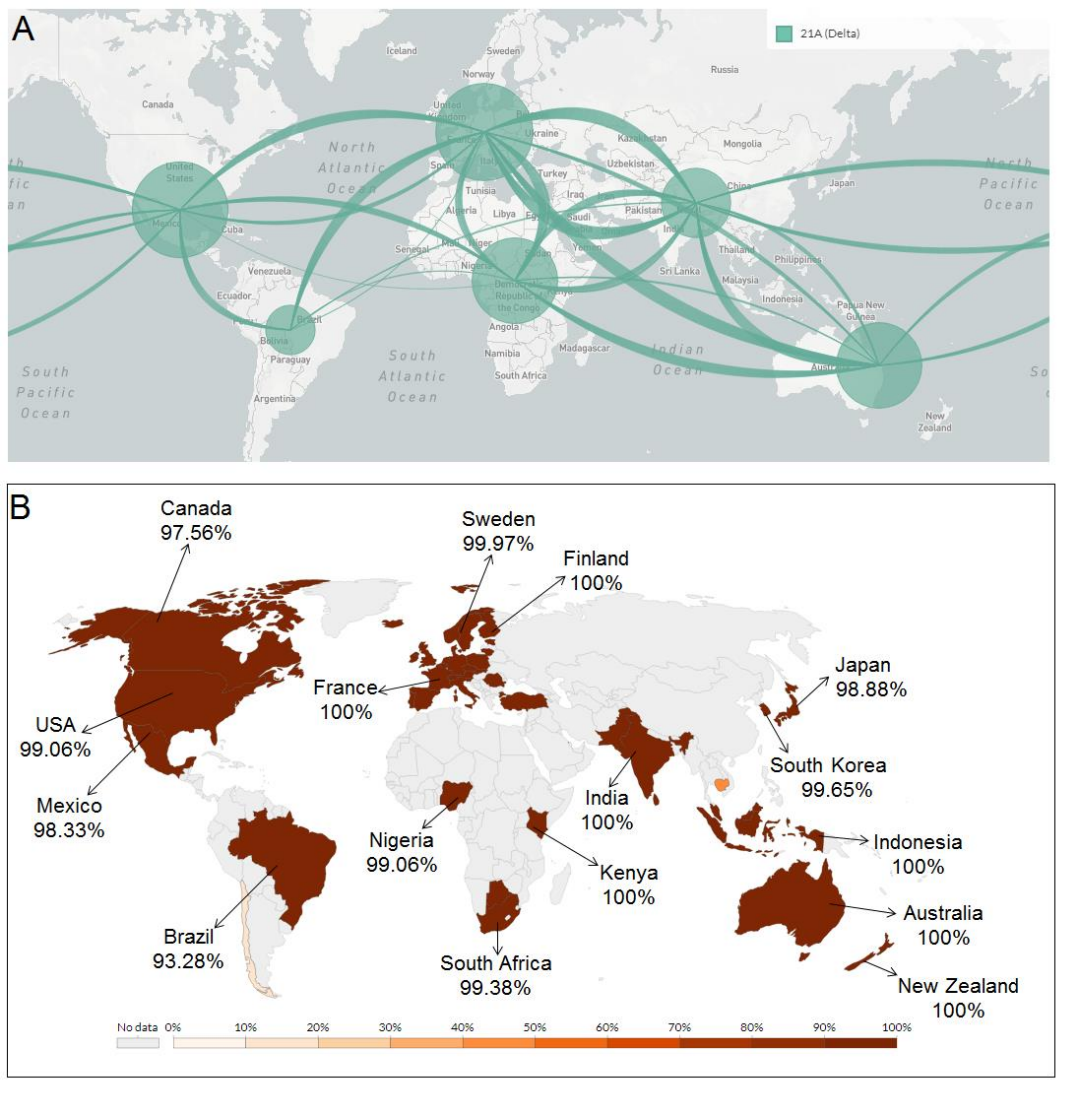
Transmission pattern and distribution pattern of delta variant throughout the world. (A) The transmission pattern of delta variants throughout the world. It demonstrates that delta variants circulated throughout the world. (B) Distribution pattern of delta variants across the globe. The frequency of the delta variant is mentioned below the country name. The distribution pattern of the delta variant illustrates the presence of the delta variant in the different parts of the world with high frequency.

The delta variant originated from India in October 2020 and subsequently spread to the UK and USA. On November 9, 2020, the variant was reported in the UK (0.01% frequency), Spain (0.28% frequency), and India (3.17% frequency). Likewise, on November 23, 2020, the variant was observed in the USA (0.01% frequency) along with the UK (0.01% frequency), India (2.94% frequency), Spain (0.25% frequency) (Supplementary Fig. 1B). On investigating the variant's transmission pattern, we found that the variant was transmitted to Europe, different parts of the USA, South Africa, Latin America, and Australia (Fig. 11A). Also, the geographical distribution pattern of the delta variant has been shown in more detail. The data showed it was distributed in the countries like the USA, Canada, Mexico, Brazil, South Africa, UK, Sweden, France, Finland, India, Japan, South Korea, Indonesia, Australia, New Zealand, etc. (Fig. 11B).

### The haplotype of delta variant

**Figure 12.**
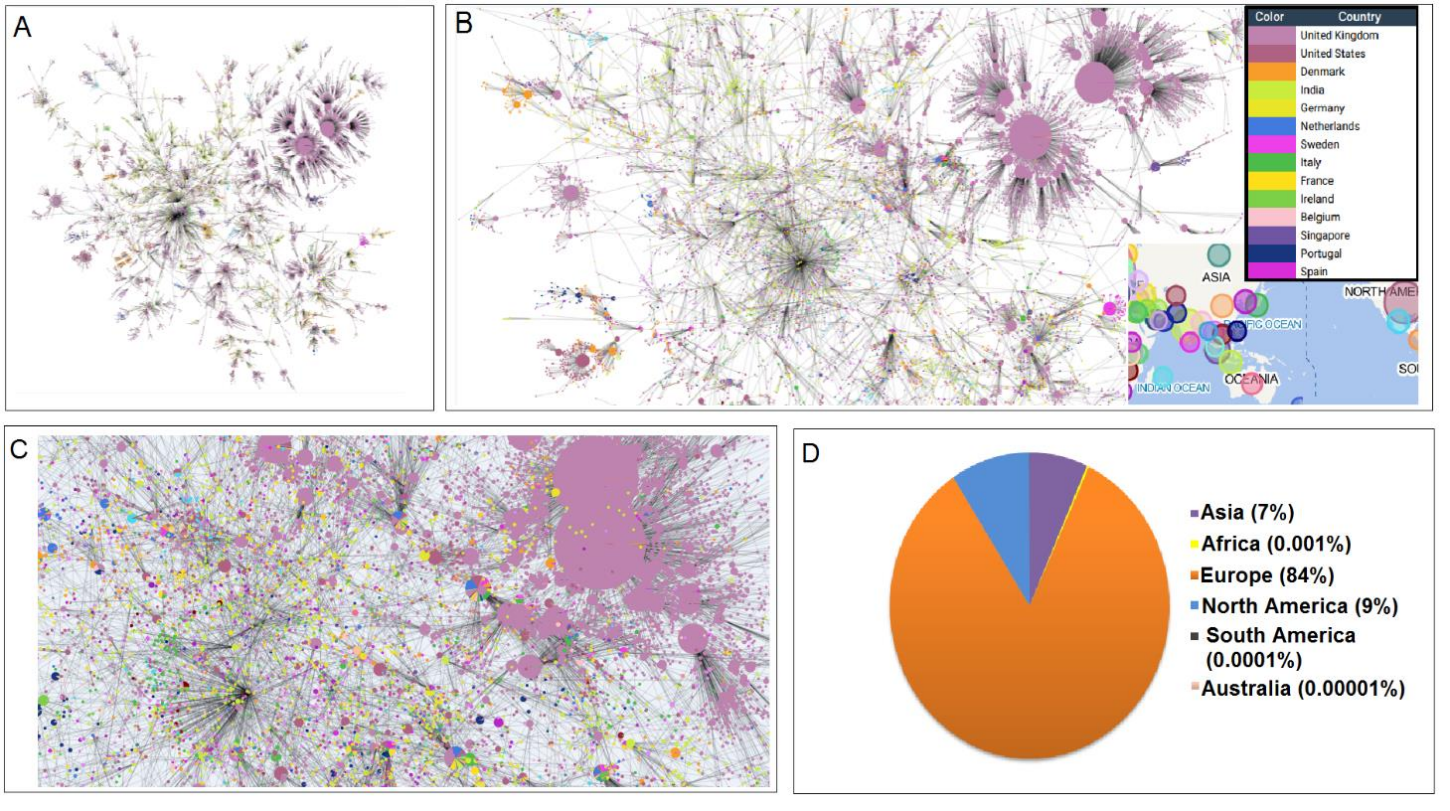
Haplotype network maps of delta variant. Every node in the depicted network represents a haplotype delta variant. (A) A haplotype network map shows the entire landscape of the inheritance of mutations of delta variants genome throughout the world. The study identified 123,526 haplotypes as the whole landscape of delta variants. (B) Country/location-wise haplotype network map shows the distribution (country-wise) of delta variant. It informs that the highest haplotype was identified from the UK (81,991). (C) An enlarged part of the large clusters from the haplotype network map was depicted again to capture the delta variant's surroundings. (D) Continent-wise haplotype distribution of the variant. It shows the percentage of haplotype distribution of delta variants in all the continents.

A delta variant haplotype network can help to understand the diversity of sequences and their clusters in the sequences of delta variants. It also might expose the unique sequence pattern of the variant. The starting node informs us about the reference sequence haplotype. The entire haplotype network model was generated to show the landscape of mutation. This haplotype network with the initial node showed its relationship with other nodes. Therefore, the haplotype network model depicted the inheritance of mutations during the generation of delta variants. About 123,526 haplotypes were identified (Fig. 12A), and our study analyzed the location-wise distribution of 21A (Nextstrain clade) or G/478K.V1 (GISAID clade), resulting in two distinct clusters in the haplotype network. The maximum haplotypes identified from countries were as follows: UK (81,991; Fig. 12B), USA (10,174), Denmark (5,637), India (4,089), Germany (2,350), Netherlands (1,899), Sweden (1,791), Italy (1,720), France (1,293), Ireland (1,257), Belgium (1,207), Singapore (1,193), Portugal (1,184) and Spain (1,133). Our analysis revealed several small clusters, and an enlarged cluster was depicted to capture the surrounding information (Fig. 12C). Europe had the highest haplotype (84%), and Australia had the lowest (0.00001%) (Fig. 12D). The haplotype network model was generated using 4308818 sequences collected worldwide on October 2, 2021. This network links all nodes (genotypes), and this visualizes the shortest set of connections. In this network, each connection length signifies the genetic distance [50]. Furthermore, all the haplotype networks informed us about the dominance of the delta variant throughout the world.

### Scatter plot model showing the dominance of delta variant

**Figure 13.**
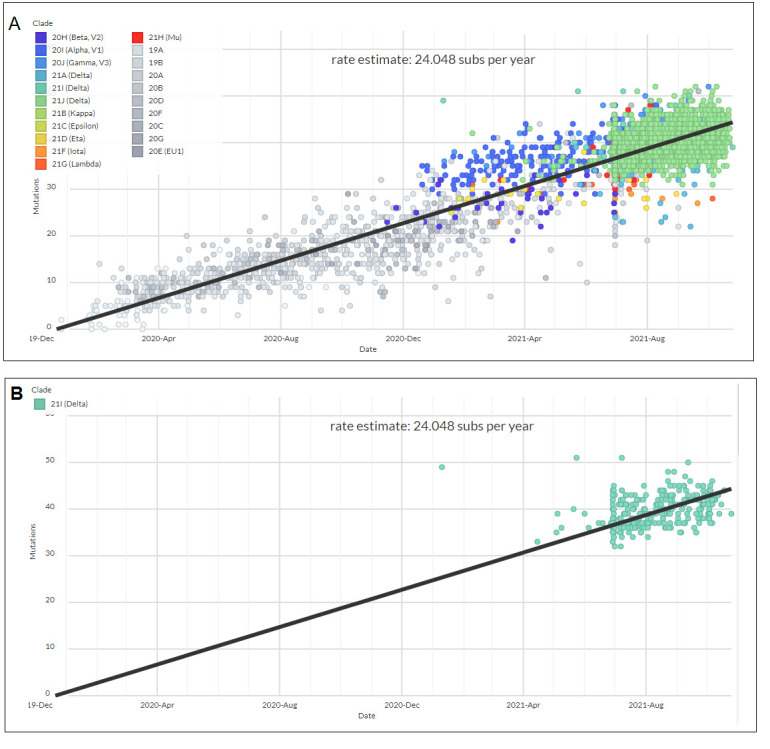
A model showing the scatter plot with a regression line of all lineages between December 2019 and August-September 2021, using only delta variant samples. (A) A model illustrating a scatter plot with a regression line of all lineages between December 2019 and August-September 2021. The Scatter plot informs that the delta variant samples are distributed in the upper position of the regression line. (B) A model illustrating a scatter plot with a regression line only using genome samples of the delta variant.

A scatter plot model was generated with a regression line using all lineages between December 2019 and September 2021. It showed how delta variants are clustered in the upper position of the regression line (Fig. 13A). The linear regression produced an estimated rate of mutation, which was 24.048 substitutions yearly. Another scatter plot was generated with a regression line using only delta variant samples, which inform us of the distribution of the delta variant along the side of the regression line and its accumulation of substitutions over time (Fig. 13B). This provides further opportunities to analyze the frequency of compensatory mutation and the clustering of intragenic mutations [51]. It will also estimate the mutation rate per variant, and the mutations are an essential source of the genetic novelty of an organism [52]. Moreover, more information can be revealed to understand the divergence times of any SARS-CoV-2 variants by using molecular clocks. As shown above, our study has observed significant mutations among delta variants (Supplementary Table 2).

## DISCUSSION

Scientists have observed that the mutation is widespread in SARS-CoV-2. Such mutation in the S-glycoprotein with the N501Y was observed from October to November 2020 in the UK. Here two new lineages of the virus were reported [53]. Kim et al. noted the D614G mutation during early 2020, affecting immunogenicity and antigenicity [54]. However, phenomena such as augmented structural/non-structural protein stability, receptor binding affinity, and immune evasion are common in both VOC and VOI variants. For example, Upadhyay et al. argued about the role of natural selection among the variants [55]. There were reports of COVID-19 infection among several vaccinated individuals even after the second dose of the BNT162b2 mRNA vaccine [56]. Also, several reports showed the infection, re-infection, immune escape, antibody escape, and vaccine escape phenomenon involving delta variants [27, 57, 58].

**Figure 14.**
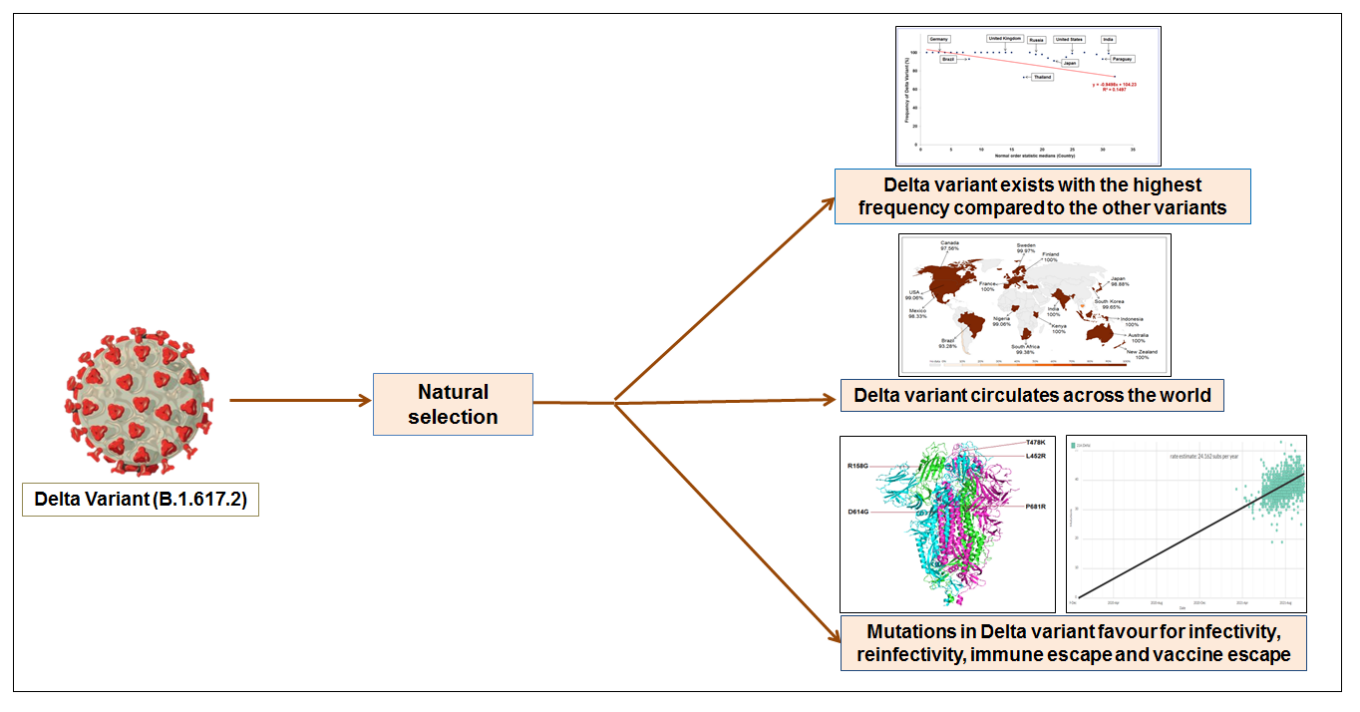
The hypothetical figure exemplifying the natural selection of the delta variant. Our previous investigations have concluded that the delta variant is occurring with the highest frequency in every country presently and circulating throughout the globe.

The key mutations like T478K, L452R, E484Q, D614G, and P681R lead to the characteristics like augmenting ACE2 binding, stability of the binding, and immune escape. It increases the biophysical fitness of the delta variant and adaptive evolution that helps to increase the viral survival and spread. In turn, the delta variants gained these properties due to natural selection in the form of rapid mutations. However, Rochman et al. stated about the role of positive selection of SARS-CoV-2 variants during the emergence of regional diversification [59]. Others have also reported positive selection among the seasonal influenza virus (ssRNA virus) [60, 61]. Furthermore, a positive selection of SARS-CoV-2 was reported by MacLean et al. where they found an adaptive evolution favoring CpG suppression in the lineage associated with nCoV clade [62]. Our study noted that the spreading of the delta variant happens due to natural selection, and the insignificant mutations in S-glycoprotein and other parts of the genome appear to catalyze the natural selection process favoring the variant (Fig. 14).

For country-wise frequency analysis and the demographic-specific composition of SARS-CoV-2 variants, our study observed high occurrences of delta variants, demonstrating the supremacy of this prominent variant. This was corroborated by an increase of delta variants in the UK [63]. Our study observed the signature shifts in the composition of SARS-CoV-2 variants and a high rise in the frequency of delta variants. The variant frequency rose to 100% in several countries, indicating the evolutionary fitness involved in the natural selection of the variant. The country-wise haplotype evaluation of the delta variant showed the haplotype signature with the highest count in the UK. Garvin et al. have developed a SARS-CoV-2 haplotype model using 15,789 full-length genomes from different variants, and they showed three major clades in the mutation clusters [10].

It has been noted that the basic reproduction number (Ro) is a significant parameter for infectious diseases, and several models have been developed to measure Ro for infectious diseases. The development of this model can help in designing the strategies for containing the disease [64]. Ro denotes the average number of infections that an infected individual can transmit to an entirely new population. The population should not be pre-exposed to any therapeutics or vaccines [65,66]. It has been observed that the Ro value of the delta variant is greater than the wild strain. It was noted that the Ro value of the wild strain is about 2.79. At the same time, the Ro value for the delta variant is approximately 5.08 [67]. However, Zhang et al. performed an analysis to calculate the transmission dynamics for the Delta variant in the Guangdong area, China and reported the Ro value to be 3.2 [68]. Due to the more infectious properties of the delta variant, the variant can contribute to its quicker transmission property (higher Ro value) compared to the wild type.

## Conclusion

The demographic haplotype destitution of delta variants with the maximum haplotype was noted in Europe as it played a major hot-spot for the molecular evolution of delta variants. Our study emphasized the paradigm shift of variants and the increase of delta variants worldwide. The evidence strongly supports the natural selection event propelling the delta variants to prosper during the pandemic. The results presented in this paper will help the health community formulate region-wise vaccination strategies to control the COVID-19 pandemic and prepare for future pandemics. However, more long-term studies are needed on delta variants to develop the best possible therapeutic against and identify the factors contributing to the evolution of the variants.

## Data Availability

The data used to support the findings of this study are included within the main text or the supplementary materials.

## Supplementary Materials

The Supplementary data can be found online at: www.aginganddisease.org/EN/10.14336/AD.2021.1117.


