# Supplementary material for: A Paradigm Shift in the Combination Changes of SARS-CoV-2 Variants and Increased Spread of Delta Variant (B.1.617.2) across the World
Source: Aging Dis. 2022 Jun 1;13(3):927–42. doi: 10.14336/AD.2021.1117 (PMC9116911; doi:10.14336/AD.2021.1117)
Supplement: Supplementary file 1 [file AD-13-3-927-s.pdf]

SUPPLEMENTARY DATA

A Paradigm Shift in the Combination Changes of SARS-CoV-2 Variants and Increased Spread of Delta Variant (B.1.617.2) across the World

Chiranjib Chakraborty[1,#,*], Ashish Ranjan Sharma[2,#], Manojit Bhattacharya[3,#], Govindasamy Agoramoorthy[4*], Sang-Soo Lee[2*]

© 2021. Chakraborty C et al. Published online at http://www.aginganddisease.org/EN/10.14336/AD.2021.1117

SUPPLEMENTARY DATA

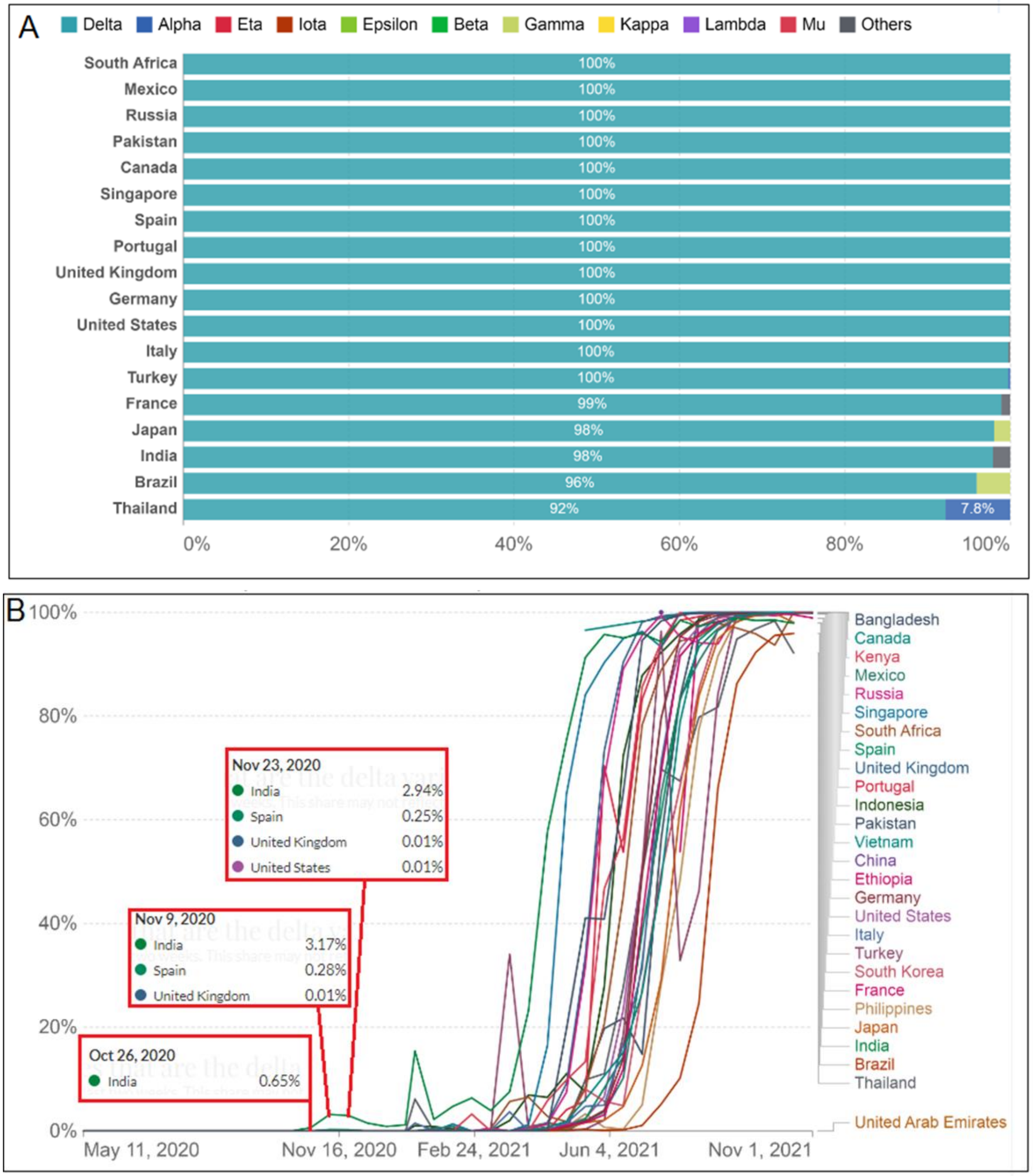

Supplementary Figure 1. The percentage share of delta sequences in the last two weeks from the considered total sample sequences. (A) The percentage share of delta from analysed sequences in the last two weeks from different countries, corresponding to each SARS-CoV-2 variant. (B) The percentage share of the delta from analysed sequences in the last two weeks. (Data source: Covariants.org and GISAID Data analysed by Our World in Data)

© 2021. Chakraborty C et al. Published online at http://www.aginganddisease.org/EN/10.14336/AD.2021.1117

SUPPLEMENTARY DATA

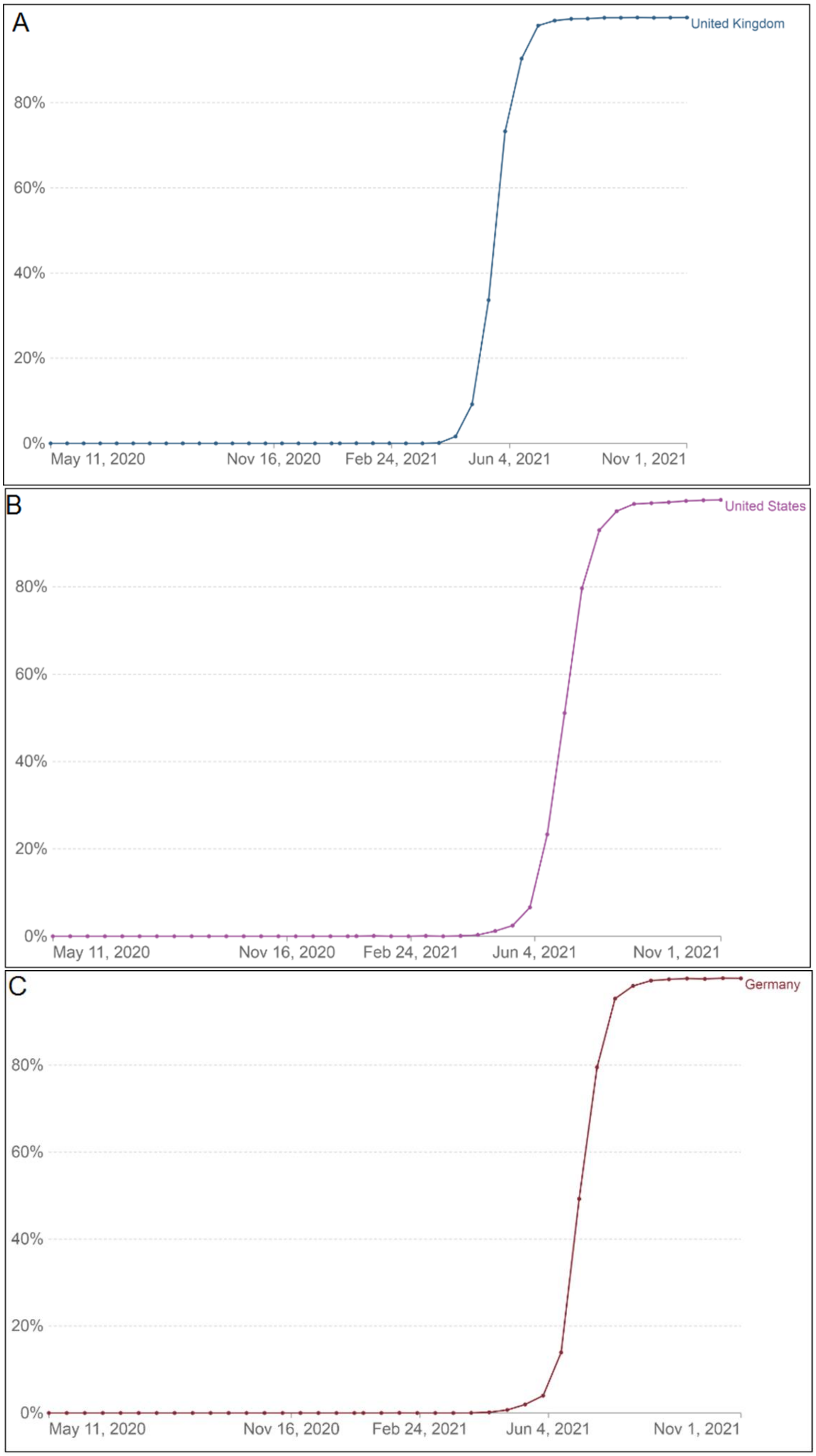

© 2021. Chakraborty C et al. Published online at http://www.aginganddisease.org/EN/10.14336/AD.2021.1117

SUPPLEMENTARY DATA

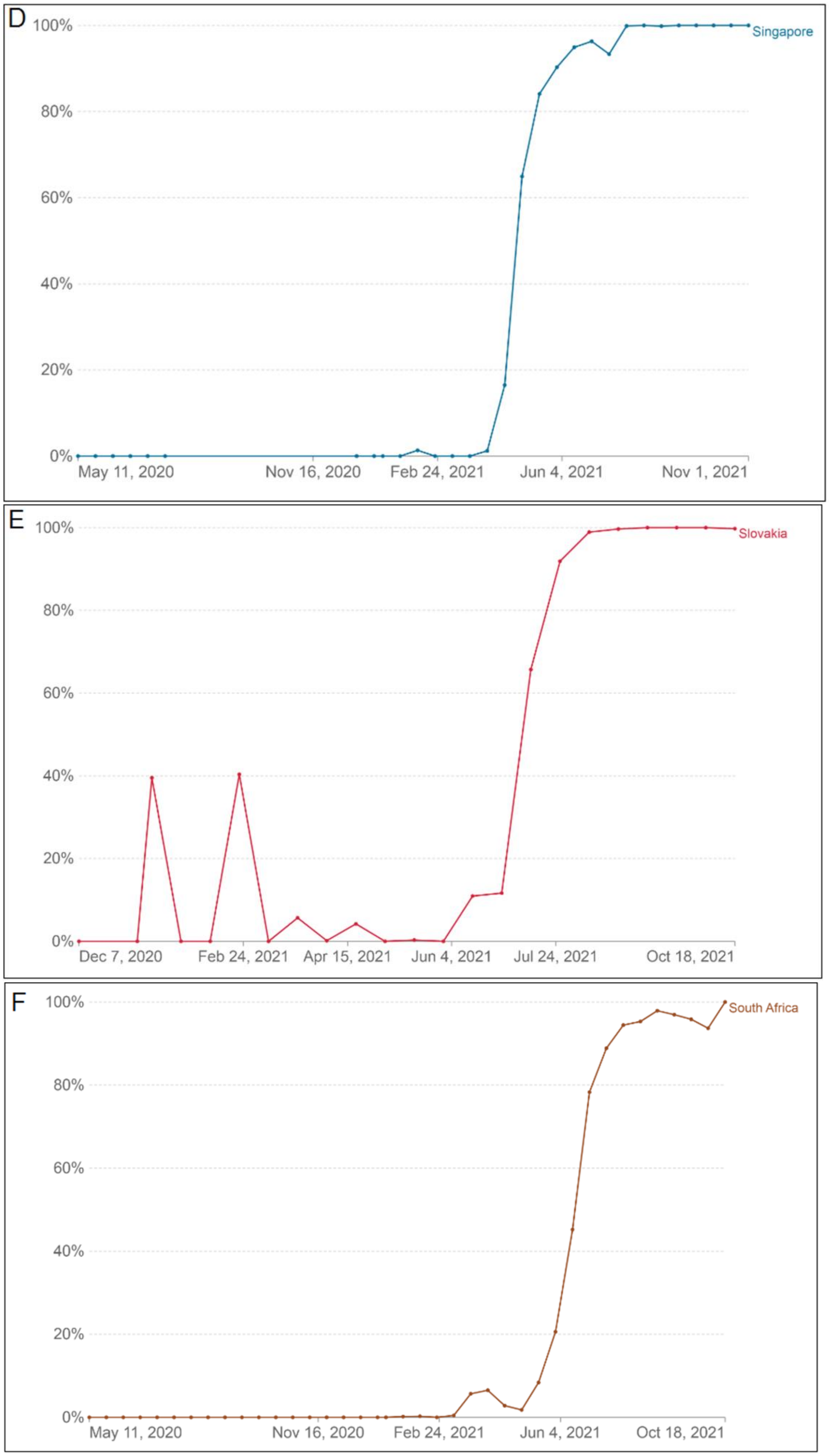

© 2021. Chakraborty C et al. Published online at http://www.aginganddisease.org/EN/10.14336/AD.2021.1117

SUPPLEMENTARY DATA

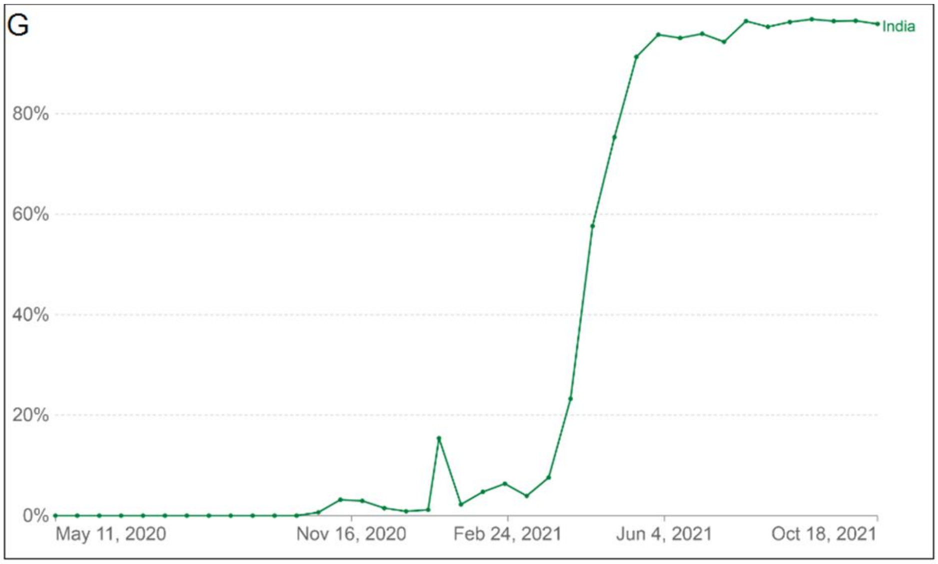

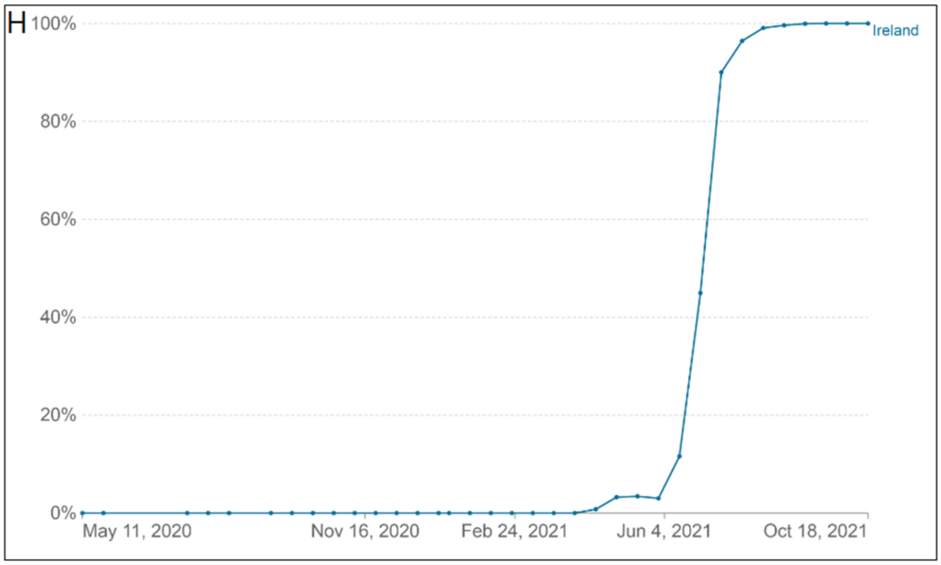

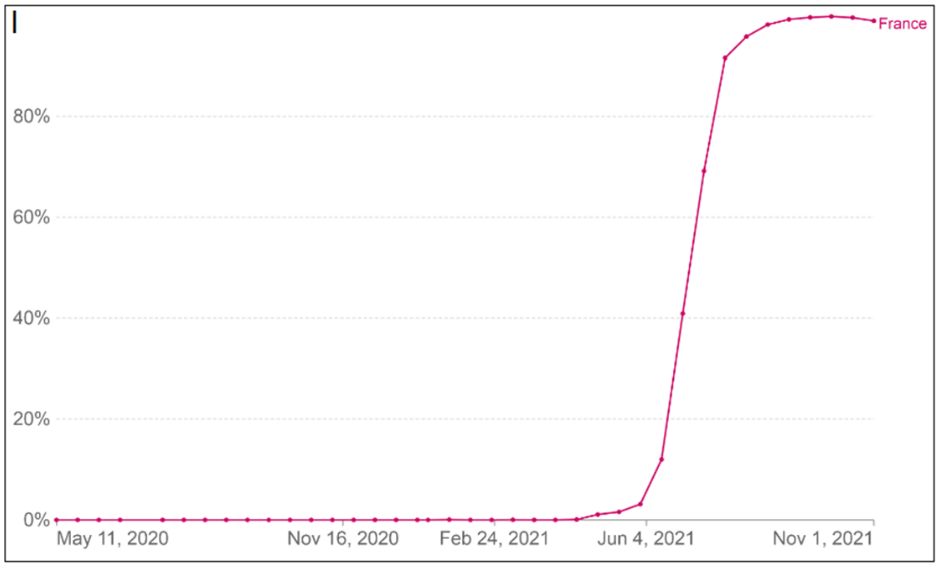

© 2021. Chakraborty C et al. Published online at http://www.aginganddisease.org/EN/10.14336/AD.2021.1117

SUPPLEMENTARY DATA

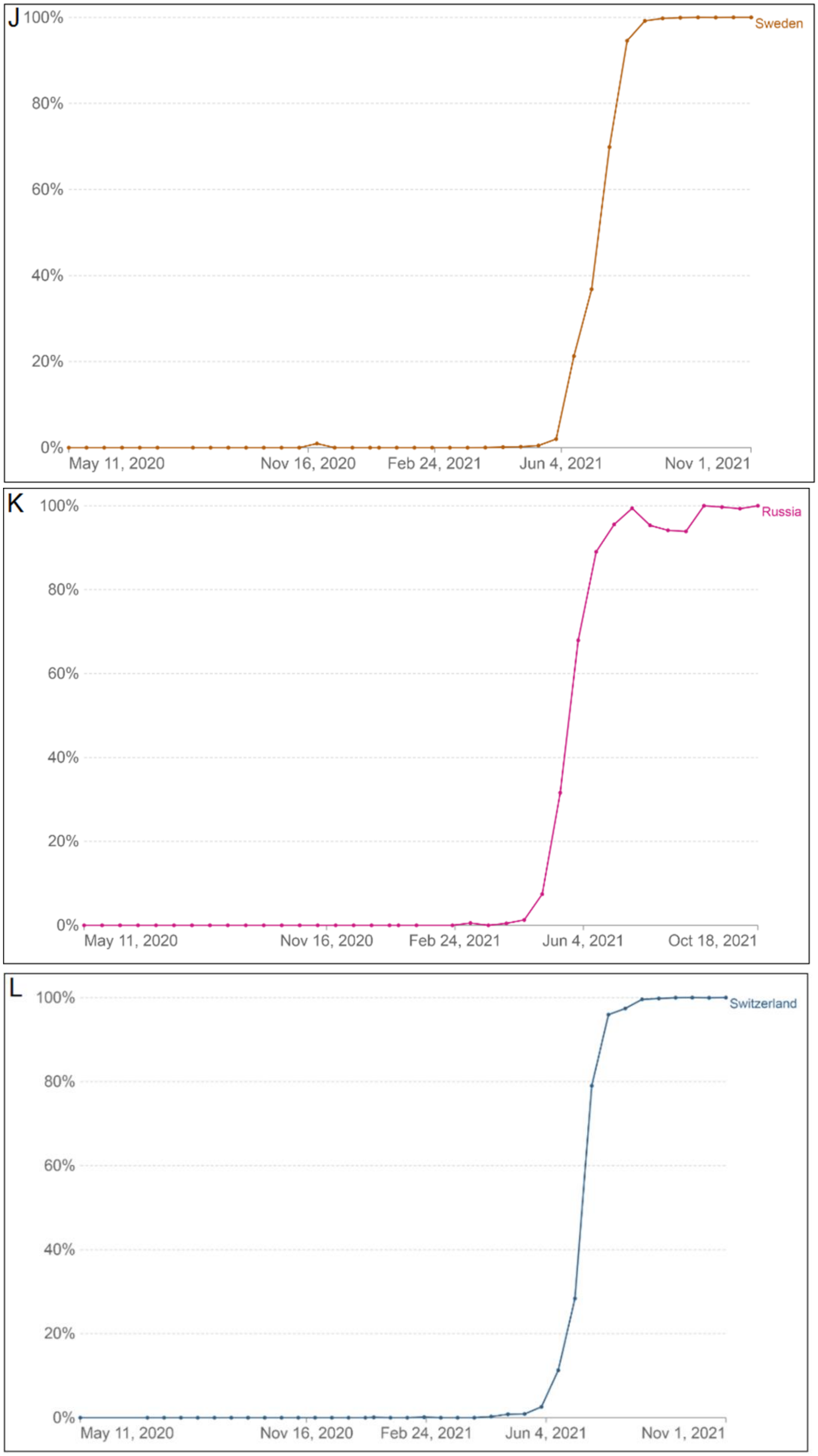

© 2021. Chakraborty C et al. Published online at http://www.aginganddisease.org/EN/10.14336/AD.2021.1117

SUPPLEMENTARY DATA

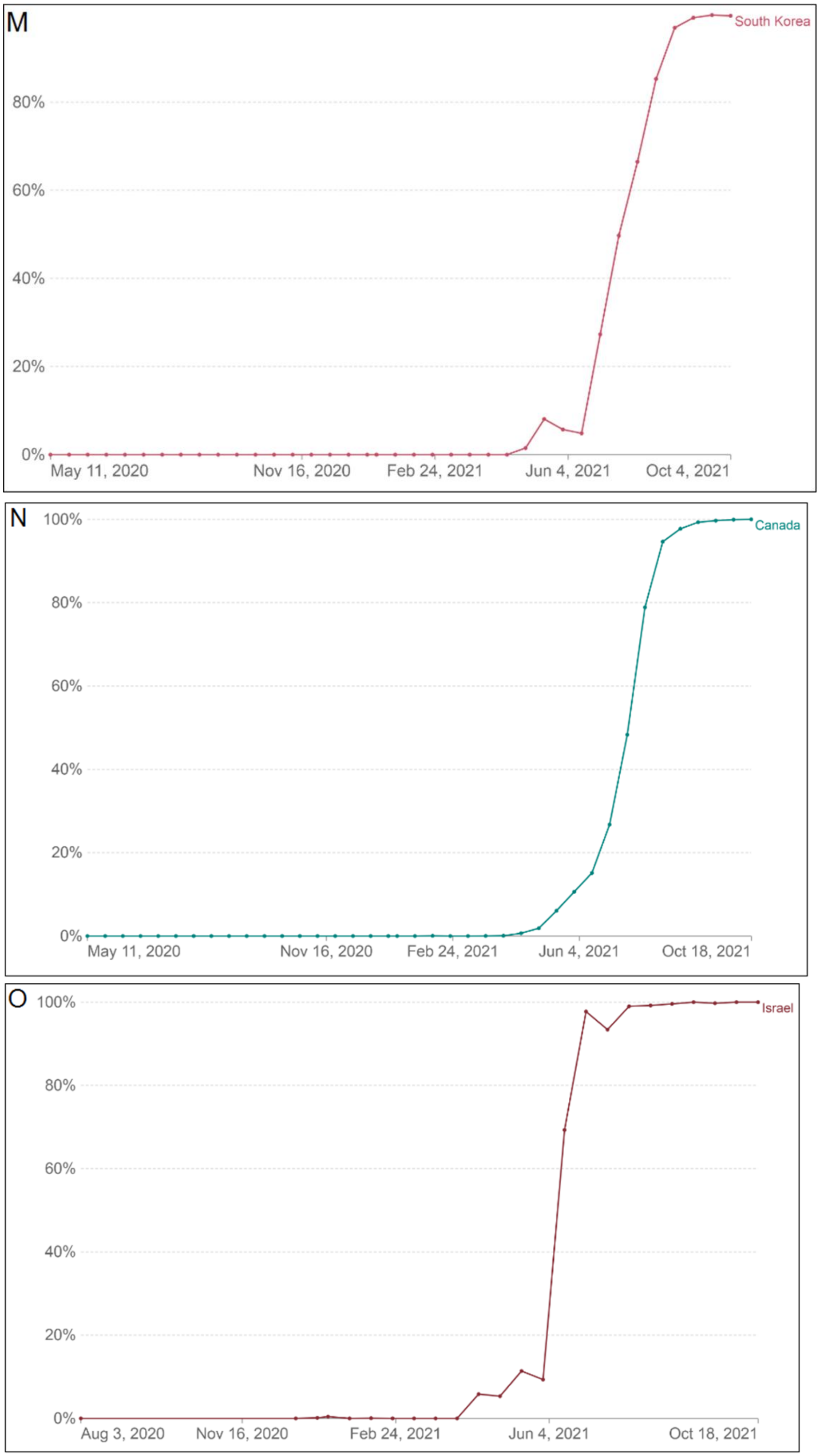

© 2021. Chakraborty C et al. Published online at http://www.aginganddisease.org/EN/10.14336/AD.2021.1117

SUPPLEMENTARY DATA

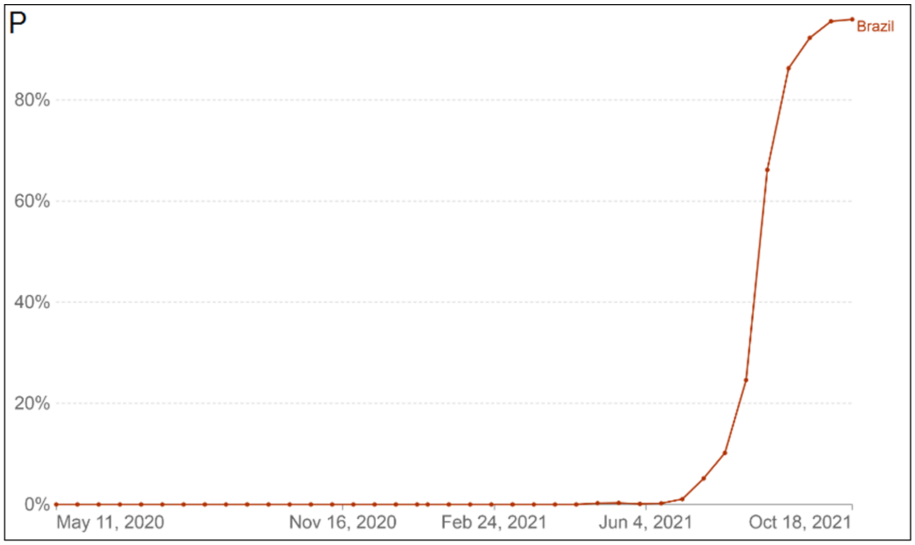

Supplementary Figure 2. Country-wise frequency of delta variants in (A) the United Kingdom, (B) the USA, (C) Germany, (D) Singapore, (E) Slovakia, (F) South Africa, (G) India, (H) Ireland, (I) France, (J) Sweden, (K) Russia, (L) Switzerland, (M) South Korea, (N) Canada, (O) Israel and (P) Brazil (Data source: Covariants.org and GISAID Data analyzed by Our World in Data)

Supplementary Table 1. Significant variants of SARS-CoV-2 and their different lineages.

Sl no.	WHO Label	Pango lineage	Nextstrain clade	GISAID clade
	Delta	B.1.617.2	21A	G/478K.V1
	Alpha	B.1.1.7	20I (V1)	GRY
	Gamma	P.1	20J (V3)	GR/501Y.V3
	Beta	B.1.351	20H (V2)	GH/501Y.V2
	Epsilon	B.1.427 and B.1.429	21C	GH/452R.V
	Eta	B.1.525	21D	G/484K.V3
	Iota	B.1.526	21F	GH/253G.V1
	Lambda	C.37	21G	GR/452Q.V1
	Kappa	B.1.617.1	21B	G/452R.V3

Supplementary Table 2. Significant mutations were observed in the different parts of the delta variant. These mutations are noted in the previous reports.

Mutations in Spike glycoprotein		Mutations in other protein
In RBD region	Other than RBD region	
L452R,<br>T478K,<br>D614G,<br>P681R,<br>D950N	T19R,<br>E156G	P2046L,<br>V2930L,<br>P314L,<br>G662S,<br>P1000L,<br>A1918V,<br>S26L,<br>V82A,<br>T120I,<br>D119I,<br>del120/121,<br>I82T,<br>D63G,<br>R203M,<br>G215C,<br>D377Y

© 2021. Chakraborty C et al. Published online at http://www.aginganddisease.org/EN/10.14336/AD.2021.1117
